# From the use of exposure index in quality control testing to the use of exposure index for quality control of clinical images

**DOI:** 10.1016/j.ejro.2022.100454

**Published:** 2022-11-09

**Authors:** Ioannis A. Tsalafoutas, Shady AlKhazzam, Huda AlNaemi, Mohammed Hassan Kharita

**Affiliations:** a Medical Physics Section, OHS Department, Hamad Medical Corporation, Doha, Qatar; b Weill Cornell Medicine-Qatar, Doha, Qatar

**Keywords:** Radiography, Quality control, Exposure index, Target exposure index, Deviation index

## Abstract

**Aim:**

The exposure index (EI) is used in routine quality control (QC) tests performed in the radiographic equipment installed in our hospitals. This study aimed at investigating the factors affecting the calculation of EI in QC and clinical images, and the implementation of target EI (EI_T_) and deviation index (DI) in clinical practice.

**Methods:**

The EI is 100 times the incident air kerma (IAK) in μGy on the image receptor, using the RQA-5 X-ray beam quality. Conformance to this relationship was investigated in QC images and clinical images acquired using anthropomorphic phantom body parts and different examination protocols, tube potential settings and radiation field sizes. Furthermore, a survey on EI_T_ and DI data from clinical images was performed.

**Results:**

Though automatic exposure control (AEC) systems have been adjusted for an IAK of 2.5 μGy, for most anthropomorphic phantom images the EIs were far from 250, depending on the manufacturer, the anatomy imaged, and the examination protocol. Regarding the survey results, DI calculation was feasible in only 38 % of the systems, since for the rest EI_T_ values have not been set. However, the rationale based on which EI_T_ have been selected is unclear. Some systems use only one while others many different EI_T_ values.

**Conclusion:**

Before using EI for quality control of clinical images image all receptors and AEC systems should be properly calibrated. Then, the methodology of selecting appropriate EI_T_ should be refined, since the EI calculation may vary, depending on the manufacturer, the anatomy imaged, and the examination protocol.

## Highlights

• Conformance to the AAPM recommendations regarding EI, EI¬T and DI was audited.
• Anthropomorphic phantoms and flat fields were used for testing EI calculation.
• Large differences were observed in clinical EI values for different examinations and manufacturers.
• EI_T_ and DI values have been found in only 38 % of the radiographic systems surveyed.

## 1 Introduction

Digital radiography images are automatically adjusted for latitude and contrast, and therefore problems with over- or under-exposed images, which were very common in screen/film radiography, no longer occur. The image ‘blackening’ is not affected by the incident air-kerma (IAK) to the image receptor. However, the noise is affected, and the higher the IAK is, the lower the noise and the better the image quality are [1]. Due to this, a trend towards increased patient dose was observed during the first years of application of digital imaging (using storage phosphor plates), which was reported as the ‘exposure creep’ [2], [3], [4].

To deal with this problem, all major manufacturers of computed radiography (CR) and digital radiography (DR) systems devised an indicator, referred to as exposure index or exposure indicator (EI), to inform the users about the IAK on the image receptor, and alert them in cases of over- or under-exposure [1]. However, the problem was that each manufacturer devised its own method to calculate the image receptor exposure. Many different definitions of EI were used, some of which were complicated and not easy to use in the everyday clinical practice. For example, depending on the manufacturer, an increasing IAK could result to a higher or a lower EI value.

This problem was eventually recognized, and pressure was exerted on all manufacturers to agree on the use of a single, universal EI. This pressure became official with the publication of a new IEC standard regarding EI [5]. According to the new definition, EI is 100 times the IAK (in μGy) on the image receptor, when an X-ray beam with certain characteristics is used (RQA-5: 70 kV, HVL = 6.8 mm Al). In this IEC document, the definitions of target EI (EI_T_) and the deviation index (DI), presented in the next section, were also introduced.

In Hamad Medical Coroporation (HMC) the new quality control (QC) program implemented since 2020, has included the routing QC testing of EI: a) in terms of displayed EI calibration accuracy using the RQA-5 beam quality, b) as an index of the correct operation of the automatic exposure control (AEC) system. Regarding the AEC QC tests, tube potential values in the range of 70–125 kV, and absorber thicknesses in the range of 1–3 mm Cu are used. Though it is known that EI is affected by the tube potential and the absorber thickness [6], it is considered that EI values in the range 200–400 are indicative of an adequately adjusted AEC system.

In routine QC tests, cases of off-limit deviations of EI calibration accuracy and/or problems in AEC adjustments were observed in X-ray systems from all manufacturers. The observed deviations suggested that the EI values of clinical images would also be affected, and this should also lead to inaccurate values of DI values, which however have never been surveyed and used as a QC tool before.

This was our motivation to revisit all the procedures regarding the EI calibration, examine which factors may affect its value in QC and in clinical conditions, and finally assess what is the level of implementation regarding the recommendations on the use of EI_T_ and DI in clinical practice.

## 2 Materials and methods

### 2.1 Background on EI, target EI and DI definitions

According to the IEC Standard 62494-1 Ed. 1 2008-08 [5] (henceforth referred to as IEC standard), the EI is defined using the following equation:

(1) EI = c_0_ × g(V)

with constant c_0_ = 100 μGy^−1^ and g(V) representing the IAK in μGy at the image receptor corresponding to the values of interest (V) and obtained from an equipment-specific inverse calibration function. It must be noted that for other beam qualities, there will be deviations from this definition, since for ‘softer’ and ‘harder’ beam qualities than RQA-5, the resulting EI values are respectively smaller and larger, than those predicted by the EI defining equation [6].

This IEC standard [5] also defines the deviation index (DI) as follows:

(2) $$\mathrm{DI}=10\;\times \log_{10}[\frac{\mathrm{EI}}{{\mathrm{EI}}_{T}}]$$

where the EI is the observed EI value of a clinical image and the EI_T_ value is the target EI for specific examination which would denote that the IAK is adequate for obtaining an image of sufficiently low noise level, to facilitate a confident diagnosis. No specification is given regarding the precision of the reported DI, apart from a footnote indicating that different EI_T_ may be required for different examinations or applications.

On the other hand, AAPM Report No. 116 [7] defines the DI as:

(3) $$\mathrm{DI}=10\;\times \log_{10}[\frac{K_{\mathrm{IND}}}{K_{\mathrm{TGT}(b,v)}}]$$

with one decimal digit of precision, K_IND_ indicating the image receptor IAK and K_TGT(b,v)_ denoting target IAK explicitly defined as a function of body part (b) and view (v) [7], [8]. Both EI and K_IND_ represent the image receptor air kerma; however, EI as defined by the IEC is unitless whereas K_IND_ is in μGy. The two DI definitions give the same numerical values. A DI value of ± 1 denotes a deviation of + 25 %/− 20 % of the reported EI (or K_IND_) with respect to EI_T_ (or K_TGT_)_._

In Table 5 of the AAPM TG232 report [8], the ranges of EI_T_ values submitted by the sites participated in the relevant survey are presented for Abdomen, Chest, Pelvis and Extremity radiographs, for CR and DR systems, and for adult and pediatric patients. These ranges are so broad (e.g. for DR systems, adult patients and abdomen, EI_T_ ranges from 149 to 890), that clearly indicate that the selection of appropriate EI_T_ should be still considered as work in progress. Indeed, the American Association of Physicists in Medicine has recently formed a task group to work on this issue (AAPM TG368: Methodology for Establishing Exam-Specific Target Exposure Indices in General Radiography).

### 2.2 Background on EI calibration practices

In HMC hospitals, radiography systems from three different manufacturers are installed, namely Siemens Healthineers (fixed and mobile), Philips medical Systems (fixed and mobile) and Fujifilm Healthcare (mobile). For the routine QC testing of the EI calibration, an RQA-5 X-ray beam quality and a radiation field size covering the whole image receptor area are employed. Five exposures are performed with different tube loadings (mAs), which result in IAK values in the range of about 0.5–25 μGy. A limit of ± 20 % for the maximum deviation of the displayed EI from the expected value according to Eq. (1) has been adopted, that is from the value resulting when multiplying the IAK value (in μGy) at the image receptor level by 100 [9]. The EI testing procedure was applied in about 100 X-ray systems (the majority of which is using the new EI), and off-limit deviations were observed in X-ray systems from all manufacturers. For Philips and Fujifilm systems, when EI calibration was off-limits, the situation was straightforward since the field service engineers promptly recalibrated the EI. However, for Siemens’ systems the situation was more complicated, since one of the displayed EI was systematically overestimated by about 50 %.

The reason why Siemens’s systems presented this trend is attributed to official EI calibration procedure. Modern Siemens X-ray systems use two different EI values; one named physical EXI (henceforth referred to as EXI_P_) and one named clinical EXI (henceforth referred to as EXI_C_). In older Siemens’ systems where only one EXI was used, this was equivalent to EXI_P_. EXI_P_ does not strictly follows the IEC standard and it is used only for calibrating the EXI_C_, which follows the IEC standard (for an RQA-5 X-ray beam quality and an IAK of 2.5 μGy on the image receptor, EXI_P_ is expected to be equal to 378 and a calibration factor is used so that EXI_C_ for 2.5 μGy is 250). In the new systems, the use of EXI_P_ has been restricted and the preset adjustment was to display only the EXI_C_; the option to display the EXI_P_ had to be activated in the settings menu. However, even when the display of EXI_P_ was activated, only the EXI_C_ is stored in the DICOM header data.

According to the Siemens system operator manual [10], EXI_P_ is a better choice for images derived in the context of constancy QC tests, while EXI_C_ is more appropriate to use in clinical images, because it considers the image content. Siemens field service engineers suggested the use of EXI_C_ instead of EXI_P_ for AEC and EI calibration QC tests, even though EXI_P_ is a better choice for QC tests [10]. However, after these arguments were communicated to a Siemens specialists’ group, permission was granted to calibrate EXI_P_ in one X-ray system according to IEC standard [5].

### 2.3 Acquisitions with flat fields

To investigate the impact of EI calibration, the examination protocol (post-processing algorithm) and the collimation (actual and virtual collimation) on the EI of QC images, a series of images were acquired using a Siemens Ysio G2 system (Siemens Healthineers, Munich, Germany), henceforth referred to as system A, where both EXI_P_ and EXI_C_ were calibrated according to the IEC standard, and a Philips Digital Diagnost system (Philips Medical Systems B.V., Eindhoven, Netherlands), henceforth referred to as system B, where the EI is reported as EI_s and it is calibrated according to IEC standard. The image receptor sizes were 43 cm × 43 cm for both systems, and the AEC operation in all these radiographic systems had been previously tested and found to be satisfactory (maximum deviations within ± 20 %), in terms of repeatability, AEC cell balance, and target IAK adjustment.

All exposures were performed using the RQA-5 beam quality (the tube potential was set at 70 kV and filter of 0.6 mm Cu positioned at the collimator assembly racks), with the central AEC sensor activated and adjusted so that an IAK of 2.5 μGy is delivered on the image receptor. Images with a radiation field smaller than that required to cover the whole field of view (flat field), with and without a dosimeter within the field of view were acquired, to investigate the effect of the unexposed pixels on the EI values, with and without the use of virtual collimation (masking). Various examination protocols were used (Chest PA, Ribs AP, Thoracic Spine AP, Abdomen AP, Pelvis AP, Skull AP, and Hand AP).

### 2.4 Acquisitions with anthropomorphic phantom body parts under AEC mode

**Fig. 1:**
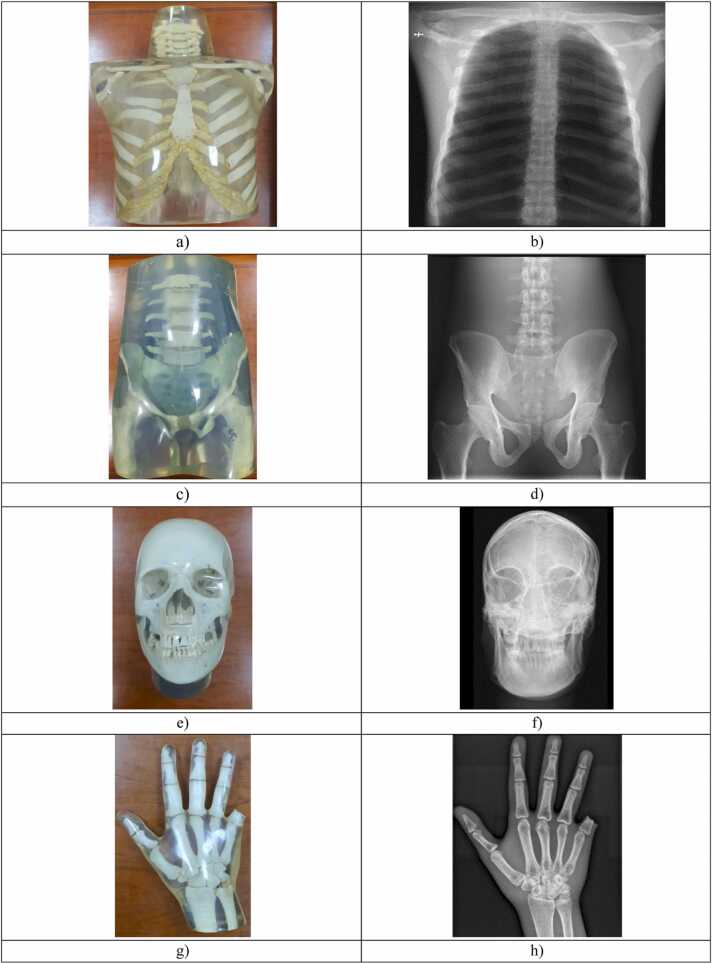
Photographs and radiographic images of the anthropomorphic phantom parts used in this study: a) Chest, b) Chest AP, c) Abdomen-Pelvis, d) Pelvis AP, e) Head, f) Skull AP, g) Hand, h) Hand AP.

To investigate the impact of the EI calibration method, the examination protocol (post-processing algorithm), the collimation (actual and virtual collimation), the kVp selection and the imaged anatomy on the EI of clinical images, a series of images were acquired using various body parts of an anthropomorphic phantom (QUART X-Ray QA Solutions) with all three systems, and a few selected examination protocols, with grid and AEC system activated. The examination protocols and the respective anthropomorphic body parts used are shown in Table 1. Photographs of the anthropomorphic phantom body parts and their radiographic appearance are shown in Fig. 1**a**–**h**.

**Table 1:** Examination protocols used in anthropomorphic phantom exposures, body part used and examination protocols settings (L, R and C stand for left, right and central AEC sensor respectively).

Anthropomorphic body part/examination protocol:	System A: Preset kVp, activated AEC sensors, and radiation field (cm^2^)	System B: Preset kVp, activated AEC sensors, and radiation field (cm^2^)
Chest/Chest PA	125, R + L, 42.7 × 42.9^a^, ^b^	125, R + L, 42 × 35^a^, ^b^
Chest/Ribs AP	79, C, 42.7 × 42.9^b^	66, C, 35 × 43
Chest/Thoracic Spine AP	75, C, 42.7 × 18	77, C, 24 × 43
Abdomen-Pelvis/Abdomen AP	81, R + L, 42.7 × 35	77, R + L, 35 × 43
Abdomen-Pelvis//Pelvis AP	75, R + L, 35 × 42.9	80, R + L, 35 × 42
Head/Skull AP	73, C, 30 × 24	77, C, 24 × 30
Hand/Hand PA	60, C, 24 × 18^c^	52, C, 18 × 24^c^

a The AEC system was set at + 1 dose (the set IAK is about 20% more compared to 0 setting, which was used in all other examination protocols).

b Additional filter of 0.1 mm Cu (system A) or 0.1 mm Cu + 1 mm Al (system B) was used according to the stored examination protocol.

c Preset examination protocol does not normally use AEC but preset mAs and no grid.

The above acquisitions were repeated using 70 kV (the kVp used for the EXI_C_ calibration), to remove the effect of different beam quality on the calculation of EI values. Any preset additional filters and dose (target IAK) increase settings were removed. Furthermore, in system A, the extended button option, and the left and right AEC sensor were activated, as appropriate for chest radiographs.

Finally, since each examination protocol incorporates a different preset post-processing image algorithm to enhance the visualization of certain anatomical characteristics and facilitate diagnosis, the chest body part was radiographed using 70 kV and all selected examination protocols, in order to differentiate the effect that the examination protocol may have on EI values from the effect of the imaged anatomic structures, which is affected by the examined anatomy and the collimation used.

### 2.5 Survey of EI values of clinical images

To assess the level of implementation regarding the IEC [5] and AAPM recommendations [7], [8] on the use of EI, EI_T_ and DI in clinical practice, the first large scale survey was performed in all radiographic systems installed in HMC hospitals, which are connected to the dose management system (Radiation Dose Monitor (RDM), Medsquare, France) of our organization. Data on EI, EI_T_ and DI were acquired from anonymized data of all clinical images acquired during the first trimester of 2021.

## 3 Results

### 3.1 Acquisitions with flat fields

For the exposures made in system A (0.6 mm Cu, 70 kV), six different radiation field sizes were used: one covering the whole image receptor (42.7 cm × 42.9 cm), and 5 smaller ones (40 cm × 40 cm, 30 cm × 30 cm, 20 cm × 20 cm, 15 cm × 15 cm, and 10 cm × 10 cm) were used. The results regarding the effect of radiation field size on EXIs can be summarized as follows:

When the unexposed area of the image receptor was not masked, for the first five field sizes EXI_P_ remained practically constant (240–249), even when different examination protocols were used, but for the 10 cm × 10 cm field, EXI_P_ dropped down to 124. This was expected since according to the Siemens operator manual [10], the exposed area is divided into a 3 × 3 matrix and the central matrix element is used to calculate EXI_P_. Therefore, since without masking the whole image receptor area is considered exposed, for radiation fields smaller than 14.2 cm × 14.2 cm, the presence of unexposed pixels is expected to reduce the EXI_p_. On the contrary, EXI_C_ rapidly reduced with the reduction of radiation field size from a starting value of 207 observed for the maximum field size (a thin penumbra on the edge was observed), to 17, 6, 2, 0 and 0. The abrupt reduction of EXI_C_ from 207 to 17, in response to the reduction of the field size 42.7 cm × 42.9 to 40 cm × 40 cm, suggested that the EXI_C_ values are smaller than expected, either considering that EXI_C_ is the mean or the median of all the image receptor pixels. Therefore, while EXI_C_ is supposed to account for the clinically relevant image content (i.e. the area of interest according to the IEC standard), and to eliminate non-relevant areas (like unexposed non-collimated areas), without proper masking of the unexposed areas, it is not clear which pixels are accounted as clinical relevant image content, and thus the EXI_C_ value is unreliable.

When the unexposed areas of the image receptor were automatically masked, the values of EXI_P_ did not practically change. For EXI_C_, apart from the maximum field where no change was observed, for all the rest five fields, EXI_C_ values were in the range 123–141, following a slight reduction with decreasing radiation field size. The fact that EXI_C_ was still much lower than EXI_P_ was attributed to the fact that with automatic masking, penumbral areas and a thin strip of unexposed white pixels were included in all four edges of all images. When the mask frame was made smaller to exclude these areas, all the EXI_C_ values increased (237–240) and became practically equal to the EXI_P_. This proved that for flat field images EXI_C_ is very sensitive to the presence of penumbra. Therefore, to use EXI_C_ instead of EXI_P_ for QC purposes, the whole image receptor area must be exposed or alternatively the radiation field must be carefully delineated in order to exclude from the EXI_C_ calculation the pixels of underexposed areas (e.g. the dosimeter probe’s shadow) and penumbral areas, in addition to unexposed non-collimated areas.

The respective flat field acquisitions with system B, demonstrated that the behavior of EI_s with field size and masking variations is like that of EXI_C_. That is, when masking is not used or when the masking does not exclude all the unexposed areas, then EI_s is reduced, and the presence of the dosimeter probe anywhere within the field of view also reduces the EI_s. This means that for the calculation of EI_s all the pixels included in the field area delimited by the virtual collimation are considered. However, in contrast to system A, in system B it was observed that the examination protocol affected the EI_s values of flat field images. The Chest AP and Ribs AP protocols resulted to up to 40 % larger EI_s values than the respective values obtained with the other protocols tested. More measurements with flat fields in system B, revealed that these differences were due to local non-uniformities across the image receptor area and to the presence of penumbra, which affected less intensely the EI_s value calculation for the images acquired with Chest and Ribs protocols than the images acquired with Abdomen, Pelvis and Thoracic Spine protocols. If the edges of latter images were gradually cropped, the EI_s values were gradually increasing towards the EI_s values of the images acquired with the Chest and Ribs protocol, which were not much affected by cropping.

### 3.2 Acquisitions with anthropomorphic phantom body parts under AEC mode

**Fig. 2:**
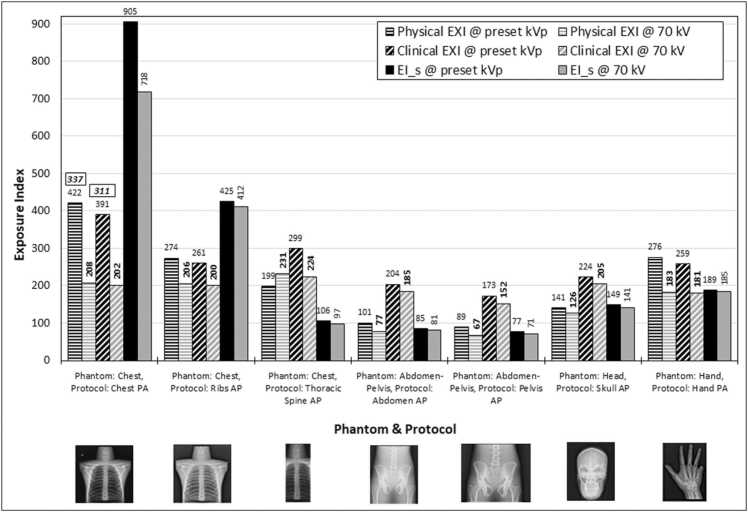
Physical EXI (EXI_p_) and clinical EXI (EXI_C_) of the images acquired with the system A with preset kVp according to the examination protocol and with 70 kV, using the respective body part of the anthropomorphic phantom, as shown in the thumbnail images under the X-axis (taken from system A). The respective EI_s values for the system B are also shown. Data labels show the exact EI value of each image (data labels within frames, are the respective values with the extended time button activated).

Fig. 2 shows the EXI_P_ and EXI_C_ values of the images acquired with system A, and the EI_s values of the images acquired with system B, using the preset examination protocols given in Table 1**,** and the respective anthropomorphic phantom body parts. Regarding the EI values of the images obtained with the preset protocols, though most images were acquired under AEC mode, it can be seen in Fig. 2 that the EXI values presented a wide variation (EXI_P_ values ranged from 89 to 422, EXI_C_ from 173 to 391, and EI_s values from 77 to 905). Given the fact that AEC is adjusted for an IAK target of 2.5 μGy, it would be expected that the EI values should be around 250. For Chest PA images where the AEC target IAK was increased by about 20 % (+ 1 dose setting), a value of about 300 would be expected.

The very high EIs observed for the Chest PA images obtained with the preset examination protocol, especially for system B, are not due to the higher IAK setting only. An additional reason is that the high kVp of the preset protocol (125 kV) combined with the reduced attenuation of the Chest body part (due to the presence of the lungs) gave rise to very low mAs values and extremely short exposure times (2 ms), which is similar to the minimum time required for the AEC system to respond and terminate the exposure (∼ 1 ms), and thus the AEC does not operate properly. Indeed, for the system A, when the Chest AP image acquisition was repeated with the extended time button activated (reduces the mA used, to increase the exposure time), the exposure time was increased from 2 to 13 ms, the mAs were reduced from 0.73 to 0.59 mA s, the EI_P_ was reduced from 422 to 337 and the EI_C_ was reduced from 391 to 311. In system B such button does not exist.

When the image acquisitions were repeated with 70 kVp, the IAK was set at 2.5 μGy for all examination protocols and the additional filters were removed for both systems, while in system A the extended time button was activated. As shown in Fig. 2, with these settings the EXI_P_ and EXI_C_ values of the Chest PA image were greatly reduced for system A, but for system B the reduction was much smaller. Furthermore, the variation in EXI_P_ and EXI_C_ values remained large, being for EXI_P_ 67–231, for EXI_C_ 152–224 and for EI_s from 71 to 718. Except for the Chest PA, for the rest images the change of kVp did not have any considerable effect on the EI values.

In the images acquired using only the chest phantom, 70 kVp and different examination protocols (to investigate whether the calculation of EI values can be affected by the post-processing algorithm), it was seen that in system A, when the maximum field was used, all clinical and physical EXI values were roughly equal. Tube loadings were ranging from 2.08 to 2.11 mA s, EXI_P_ values from 206 to 209 and EXI_C_ values from 200 to 203. Therefore, it was verified that for the Siemens systems, the different examination protocols have no effect on the calculation of EXI_P_ and EXI_C_ in clinical images.

Both EXI_P_ and EXI_C_ values were lower than 250, apparently because though the required mAs were calculated based on the attenuation of the lungs (under which the AEC sensors are located), the presence of the spinal cord and the other bony and soft tissue areas in the periphery of the image which attenuate the X-ray beam more heavily than the lungs, resulted to the respective pixels having less exposure, reducing thus the EXI_P_ and EXI_C_ values of the images [6]. When the field size was varied, both EXI_P_ and EXI_C_ also varied, because of different image content which changed the proportions of pixels with high and low exposure used for the calculation of the EXI values. In system B, the change of field size also changed the EI_s in a way similar to EXI_C,_ but for the same field size, the protocols designated for the chest anatomical area (Chest PA and Ribs AP) gave EI_s values about 3 times higher than the rest protocols. Therefore, for the system B, it was verified that the examination protocol can affect the calculation of EI values in clinical images.

### 3.3 Survey of EI values of clinical images

The results from the first large scale survey on EI using data for the first trimester of 2021, revealed from a total of 58 radiographic systems connected to the RDM, only 38 % had EI_T_ values set (11/20 of fixed Siemens’s systems, 0/5 of Siemens mobile systems, 0/25 of Philips’s systems and 8/8 of Fujifilm’s systems). For the 11 Siemens systems the situation was very confounding regarding both the number of preset EI_T_ values and the number of examination protocols to which those were applying as they were: a) two systems with EI_T_ set at 312 (in one system this value applied for 65 different protocols, in the other for a single general examination protocol), b) four systems with EI_T_ set at 250, that applied to a different number of examination protocols (114, 8, 7 and 4, respectively), c) Five systems with EI_T_ set either at 250 (for 181, 162, 158, 62 and 8 examination protocols, respectively) or at 100 (for 4, 5, 3, 2 and 2 examination protocols, respectively). It must be noted that regarding the Siemens systems, in the record kept in RDM for each radiograph, the values of EXI_C_, EI_T_ and DI were stored but not of EXI_P_. In the 8 Fujifilm systems there were identified 14 different EI_T_ values (150, 250, 300, 325, 350, 400, 500, 550, 600, 700, 780, 876, 1300 and 1500). Two of these systems had only one EI_T_ value each (300 and 876), and one had 11 different EI_T_ values that differed by a factor of 10. It should be noted the DI calculation in the case of Siemens systems was made in steps of 0.5, while in the Fujifilm systems the DI values are calculated with a precision of one significant decimal digit.

## 4 Discussion

From the measurements and from the communication with the Siemens specialists’ group, it was clarified that EXI_C_, is the median value of all pixels included in the area that is considered to comprise the clinical content. It was also clarified that EXI_P_ is used only to calibrate EXI_C_ and for this reason it is not necessary to comply with the IEC standard [5]. However, it was confirmed that if the Siemens official methodology is slightly revised, so that after the calibration of the EXI_C_ to calibrate also the EXI_P_ (using the same correction factor as the one used for calibration of the EXI_C_), then both EXI_P_ and EXI_C_ will comply with the IEC standard [5].

The use of EXI_C_ instead of EXI_P_ for QC purposes with flat fields seems incompatible with its definition, since in this case no clinically relevant image content exists. However, the results from flat fields acquisitions suggest that EXI_C_ could be used for QC purposes as well, if precautions are taken so that the collimated image area does not include any penumbral areas or areas shadowed by the dosimeter. EXI_C_ is extremely sensitive to the automatic virtual collimation and if the algorithm which detects the exposed part of the image receptor and applies masking to the unexposed part fails, even partially, then the EXI_C_ will be considerably reduced, something that may also happen in clinical images. This is in line with Herrmann et al. [11] observations, that errors during exposure field recognition can cause inaccurate DI readings (due to inaccurate EI calculation), in a way which may vary among manufacturers.

From the measurements it was also confirmed that to define the area of clinical content it is required to have the auto-masking feature activated, to exclude the collimated (unexposed) areas of the image receptor. From an additional QC image acquired in system A, with the Cu filter slightly retracted from only one side to have a peripheral band of about 5 cm on the image receptor exposed with the bare X-ray beam (a situation which is very common in clinical practice), it was seen that the EXI_P_ value was 237 and the EXI_C_ was 234, without modification of the automatic masking margins. This proved that the algorithm used to calculate EXI_C_, can identify areas of the image that are exposed with the bare X-ray beam and exclude them, since they do not represent clinical content. System’s B response to the above setting was like that of system A, proving that the algorithm used for calculation of EI_s can also detect and exclude those pixels exposed to the bare X-ray beam.

According to our notion of a true clinical EI, it would be ideal if the EXI_C_ could recognize the clinically relevant image content, that is, the anatomic areas of primary interest e.g. the lungs in Chest AP/PA images, the Ribs in the Ribs AP images, the abdomen in Abdomen AP images and the spinal cord in Thoracic Spine AP images. In principle, the AEC sensors activated are those that are covered by the anatomic regions of primary interest, which should be exposed in such a way so that the IAK reaching the activated IAK sensors is slightly more than 2.5 μGy (the AEC sensors are about 2 cm above the image receptor). Therefore, if the IAK to the image receptor is 2.5 μGy, the EXI_C_ should be about 250. Obviously, this is not the case in practice, since apart from the dependence of EXI_C_ on the X-ray beam quality (which is also affected by the patient attenuation), the AEC sensors have a fixed size and are located at a fixed position, and do not always cover only one anatomical area nor only the anatomic areas of primary clinical interest. Don et al. [6] provided examples to explain why the clinical EI cannot be the same as that expected according to the IAK settings, since the inclusion of underexposed regions within the image (e.g. the upper abdomen in a Chest PA image) or part of the surrounding overexposed area (exposed to the bare X-ray beam), may strongly affect the calculated EI, if they are recognized as relevant image content.

As can be seen in Fig. 2, different EI_T_ may be required for digital systems from different manufacturers, depending on the way that EI is defined and whether is affected or not by the examination protocol post-processing algorithm. This is an additional complication which makes the selection of proper EI_T_ a cumbersome task in institutions with radiographic systems from different manufacturers, especially when each system may have many different examination protocols. This task would be facilitated if manufacturers provided initially settings of EI_T_ which could be later revised by the users in the context of optimization. However, the results of the survey regarding the EI_T_ currently set in the radiographic systems installed in HMC, exhibited that the present situation is confounding, given the fact that some systems have no EI_T_ set, some have only one EI_T_ for many radiographic examinations, and others have many and much different EI_T_ for different radiographic examinations.

When it comes to selecting preliminary EI_T_ values, it seems reasonable to assume that a good starting point would be to select the median values of EI of all clinical images on the radiographic systems of each manufacturer, for each radiographic projection. However, it is important to stress that before setting EI_T_ for different examination protocols based on data acquired using software like RDM, care must be taken so that in each radiographic system are checked and verified: a) the correct calibration of EI, b) the correct adjustment of the AEC systems, c) the proper operation of the auto-masking procedure, d) the use of the appropriate examination protocol for each radiographic projection, and finally, e) the proper selection of manual exposure factors (and SID) for each examination protocol based on patient body habitus, for both mobile units and fixed systems when AEC is not used.

As has been reported in the AAPM reports [7], [8], the current trend is to define EI_T_ values for different radiographic projections and use the DI to indicate whether an image is acquired at appropriate dose level (regarding the IAK to the image receptor) and detect systematic over- and under-exposures. It has been stressed that EI values are not directly related to patient dose [6], since for example the use of a larger field than that required or a kVp value smaller than that required, may not be reflected on the EI values but will be spotted when a kerma are product (KAP) meter is used.

In fully digital imaging systems, when examination programs are used, the kVp values and radiation field sizes are preset. Though KAP values are expected to differ for different patient thicknesses, the EI should be roughly similar for both thin and fat patients. Images with EI values far from EI_T_ values will result in large positive or negative DI values, which will denote that something went wrong. This could happen in the case where a wrong examination protocol is used, the patient positioning is not correct, or the patient body habitus deviates much from the ordinary. However, it may also reveal drifts in the AEC operation which may be due to malfunction. The use of DI is especially useful in mobile units, where the lack of AEC may give rise to significant over-exposures [12].

Regarding the limitations of this study, it must be noted that only two radiographic systems from two manufacturers were studied, using a limited number of examination protocols and exposure conditions. Also, mobile systems were not included. In mobile systems except for the fact that AEC does not exist, when grid is not used, the X-ray beam spectrum incident on the image receptor is expected to be much ‘softer’, due to the presence of a large fraction of scattered radiation. Therefore, the EI is expected to be lower than that predicted by the EI defining equation [6], and this may also affect the selection of EI_T_ values. Therefore, the results of our study cannot be generalized to other examination protocols and different radiographic systems of the same or different manufacturers before they are validated. More measurements are required, using more radiographic systems, examination protocols and exposure conditions (including different kVp values, additional filtrations, and collimations), with and without the bare X-ray beam or penumbral areas included in the imaged areas, before concluding on the effect that each of these factors may have on the calculated EI value in different models of fixed and mobile radiographic systems from different manufacturers.

## 5 Conclusion

In this study the effect of various parameters in the calculation of EI in QC and clinical images using an anthropomorphic phantom was investigated in detail. It was determined that the anatomy examined, the collimation (physical and virtual), the tube potential, and depending on the manufacturer, the post-processing algorithm of the examination protocol, may all affect the EI value.

The first large-scale survey performed regarding EI of clinical images in our organization, exhibited the absence of EI_T_ settings in most of the surveyed radiographic systems, and deficiencies in terms of the EI_T_ value setting methodology and the subsequent problems in the DI calculation, in the rest systems. Therefore, at this point the use of EI of clinical images as a quality control metric (via the use of DI), is not yet applicable. However, the results of this study have revealed the steps that have to be taken to select appropriate EI_T_ values and enable the use of DI values as a meaningful quality metric of patient radiographs.

## CRediT authorship contribution statement

All authors substantially contributed to the conception or design of the research and analyzed/interpreted results. Measurements and survey data collection were made by IAT and SA. All authors contributed to drafting the manuscript or revising it critically for important intellectual content.

## Funding statement

No funding source, financial support or financial interest to disclose. Open Access funding is expected to be provided by the Qatar National Library.

## Conflict of interest statement

There is no conflict of interest.
